# Supplementary material for: CD1d-dependent immune suppression mediated by regulatory B cells through modulations of iNKT cells
Source: Nat Commun. 2018 Feb 15;9:684. doi: 10.1038/s41467-018-02911-y (PMC5814456; doi:10.1038/s41467-018-02911-y)
Supplement: Supplementary file 1 — Supplementary Information [file 41467_2018_2911_MOESM1_ESM.pdf]

CD1d-dependent immune suppression mediated by regulatory B cells through modulations of iNKT cells

Oleinika *et al*.

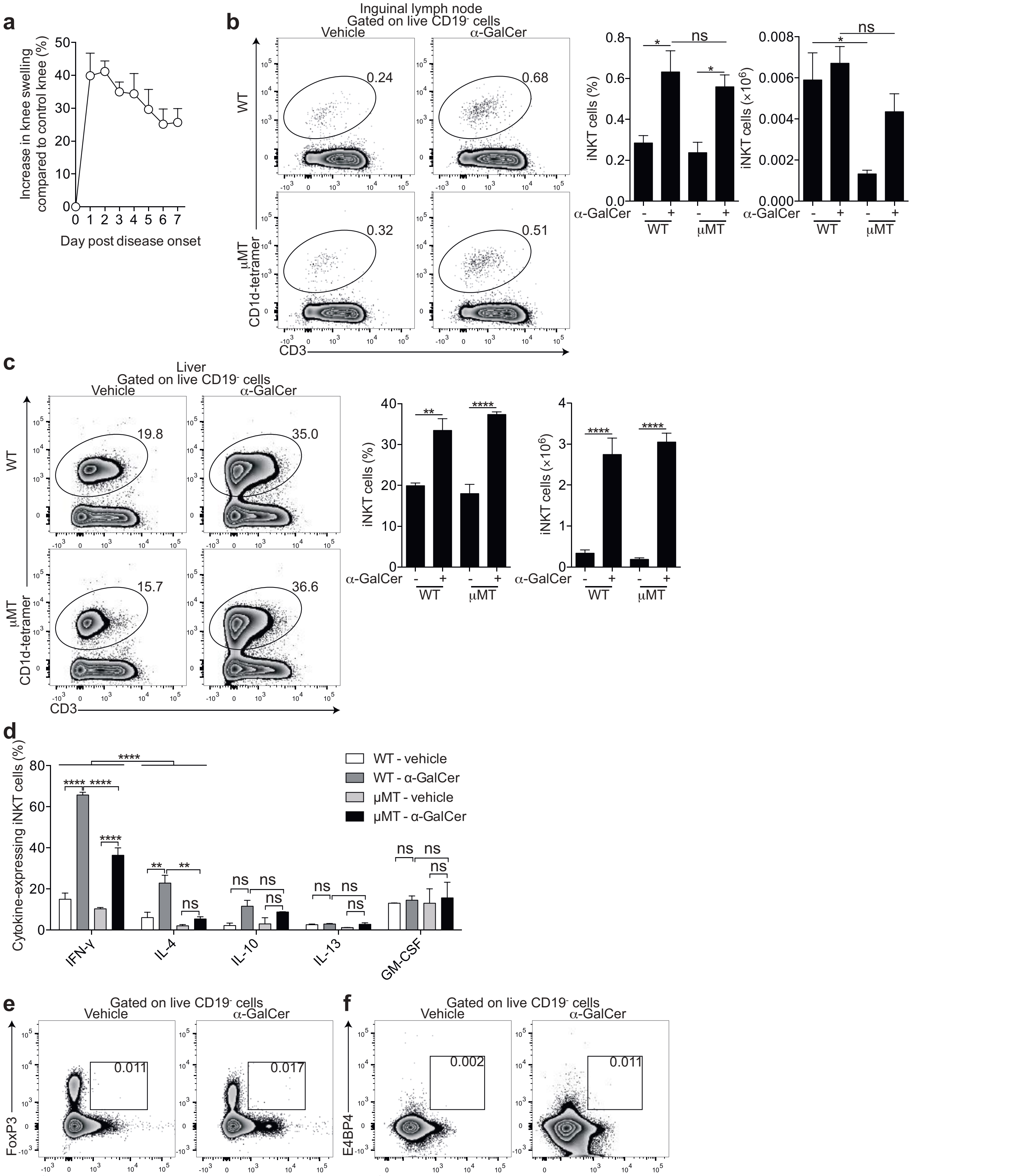

Supplementary Figure 1. B cells and α-GalCer in the regulation of iNKT cell phenotype. (a) Mean clinical antigen-induced arthritis score of WT mice. Y axis shows percentage swelling in antigen-injected knee compared to control knee (n=5). (b) Representative flow cytometry plots and bar charts showing the frequency and number of inguinal lymph node iNKT cells in μMT and WT mice that received α-GalCer or vehicle alone (n=4 per group, one of three experiments is shown). (c) Representative flow cytometry plots and bar charts showing the frequency and number of liver iNKT cells in μMT and WT mice that received α-GalCer or vehicle alone (n=4 per group, one of three experiments is shown). (d) Bar chart showing the frequency of IFN-γ$^+$, IL-4$^+$, IL-10$^+$, IL-13$^+$, and GM-CSF$^+$ splenic iNKT cells from α-GalCer- or vehicle-treated μMT and WT mice (n=2 per group, one of two experiments is shown). (e) Representative flow cytometry plots showing the frequency of FoxP3$^+$CD1d-tetramer$^+$ splenic iNKT cells from α-GalCer- or vehicle-treated WT mice (one of three experiments is shown). (f) Representative flow cytometry plots showing the frequency of E4BP4$^+$CD1d-tetramer$^+$ splenic iNKT cells from α-GalCer- or vehicle-treated WT mice (one of three experiments is shown). b, c analyzed on day 3 post-disease onset, d-f analyzed 16h post-disease onset. Data are mean±s.e.m. ns not significant, **P*<0.05, ***P*<0.01, *****P*<0.0001 (b-d one-way analysis of variance (ANOVA)).

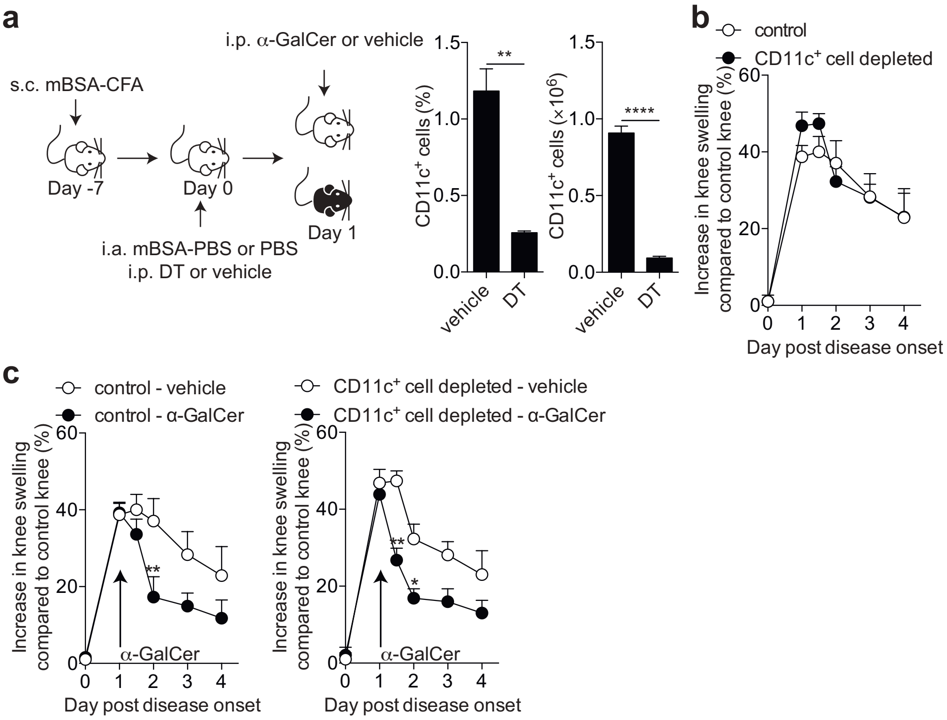

Supplementary Figure 2. Dendritic cell role in α-GalCer-mediated suppression of arthritis. (a) Left, schematic showing experimental design. Male CD11c- diphtheria toxin receptor (DTR) mice were immunized subcutaneously at the tail base with methylated bovine serum albumin (mBSA) in complete Freund's adjuvant (CFA) and phosphate-buffered saline (PBS). Diphtheria toxin (DT) was administered to deplete $CD11c^+$ cells, or PBS as a control. Right, bar charts showing the frequency and number of splenic $CD11c^+$ cells in $CD11c^+$ cell depleted (diphtheria toxin (DT) administered) and control mice prior to α-GalCer or vehicle administration (n=3 per group, one of two experiments is shown). (b) Mean clinical score of $CD11c^+$ cell depleted and control mice following induction of arthritis. Y axis shows percentage swelling in antigen-injected knee compared to control knee (n=5 per group, one of two experiments is shown). (c) Mean clinical score of $CD11c^+$ cell depleted mice (right) and control mice (left) that received α-GalCer or vehicle alone, one day following induction of arthritis. Y axis shows percentage swelling in antigen-injected knee compared to control knee, arrow indicates time of α-GalCer administration (n=5 per group, one of two experiments is shown). Data are mean±s.e.m. **P*<0.05, ***P*<0.01, *****P*<0.0001 (a Student's *t*-test, b, c two-way ANOVA).

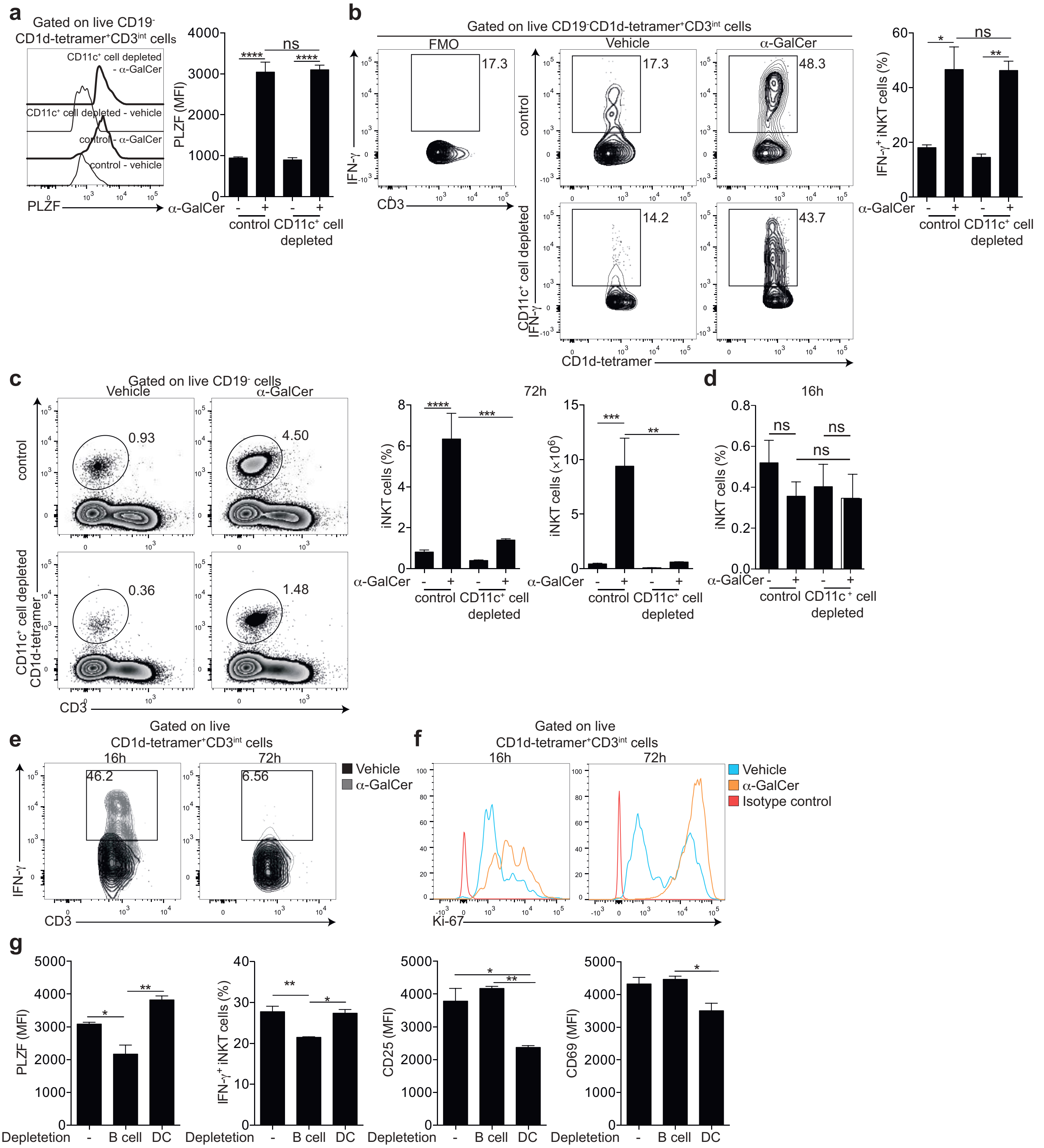

Supplementary Figure 3. Dendritic cell role in α-GalCer-iNKT cell activation. (a) Representative histograms and bar chart showing median fluorescence intensity (MFI) of PLZF in splenic iNKT cells from α-GalCer- or vehicle-treated CD11c$^+$ cell depleted and control mice (n=3 per group, one of two experiments is shown). (b) Representative flow cytometry plots and bar chart showing the frequency of IFN-γ$^+$ splenic iNKT cells from α-GalCer- or vehicle-treated CD11c$^+$ cell depleted and control mice (n=3 per group, one of two experiments is shown). (c) Representative flow cytometry plots and bar charts showing the frequency and number of splenic iNKT cells in CD11c$^+$ cell depleted and control mice that received α-GalCer or vehicle alone (n=5 per group, one of two experiments is shown). (d) Bar chart showing the frequency of splenic iNKT cells in CD11c$^+$ cell depleted and control mice that received α-GalCer or vehicle alone (n=4 per group, one of two experiments is shown). (e) Representative flow cytometry plots showing the frequency of IFN-γ$^+$ splenic iNKT cells 16 and 72h following α-GalCer administration. (f) Representative histograms showing MFI of Ki-67 in splenic iNKT cells 16 and 72h following α-GalCer administration. (g) Bar charts showing the expression of PLZF, IFN-γ, CD25 and CD69 in iNKT cells from B cell-depleted, dendritic cell-depleted, and undepleted control splenocytes following *in vitro* treatment with α-GalCer and IL-2 (n=3 per group, one of two experiments is shown). a, b, d analyzed 16h post-disease onset, c analyzed on day 3 post-disease onset. Data are mean±s.e.m. ns not significant, **P*<0.05, ***P*<0.01, ****P*<0.001, *****P*<0.0001 (A-D, G one-way ANOVA).

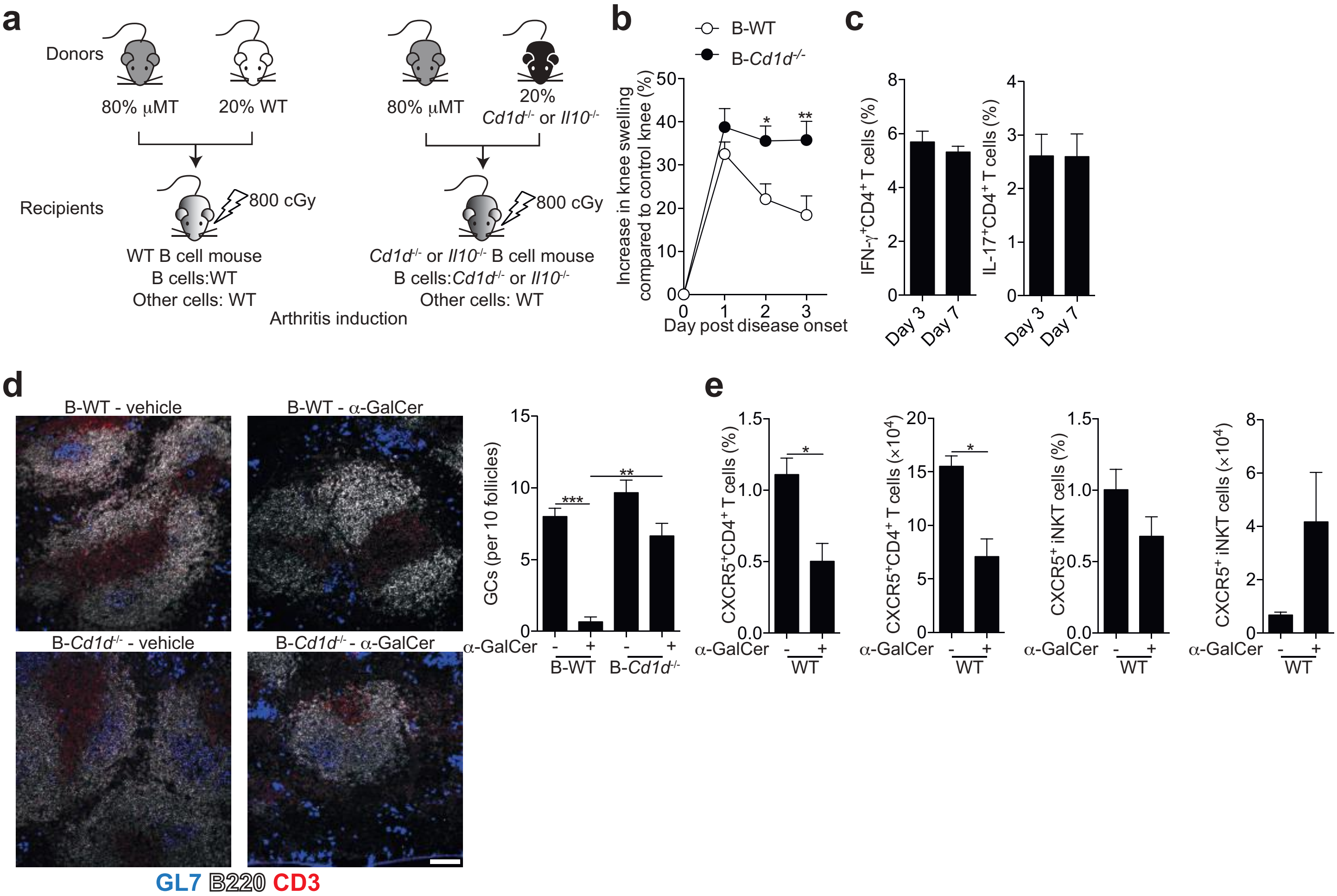

Supplementary Figure 4. Generation of mixed bone marrow chimeric mice. AIA progression in chimeric mice lacking CD1d specifically on B cells. Comparison of IFN-γ- and IL-17-expressing CD4$^+$ T cells on day 3 and day 7 following AIA induction. Germinal centers, Tfh and NKTfh cells following α-GalCer treatment. (a) Schematic showing experimental design for the generation of B-*Cd1d*$^{-/-}$, B-*Il10*$^{-/-}$ and B-WT mixed bone marrow chimeric mice. (b) Mean clinical score of B-*Cd1d*$^{-/-}$ and B-WT chimeric mice following induction of arthritis. Y axis shows percentage swelling in antigen-injected knee compared to control knee (B-*Cd1d*$^{-/-}$ n=5, B-WT n=5, one of three experiments is shown). (c) Bar charts showing the frequency of splenic IFN-γ$^+$ and IL-17$^+$CD4$^+$ T cells on day 3 and day 7 following AIA induction (n=4 per group). (d) Representative immunofluorescence and bar chart showing the number of splenic germinal centers, defined as discrete GL7$^+$ areas within B220$^+$ follicles, in B-*Cd1d*$^{-/-}$ and B-WT chimeric mice that received α-GalCer or vehicle alone. Bar, 100 μm (n=3 per group, one of two experiments is shown). (e) Bar charts showing the frequency and number of splenic CXCR5$^+$CD4+ T cells and CXCR5$^+$ iNKT cells in WT mice that received α-GalCer or vehicle alone (n=3, one of two experiments is shown). d, e analyzed on day 3 post-disease onset. Data are mean±s.e.m. **P*<0.05, ***P*<0.01, ****P*<0.001 (b two-way ANOVA, c, e Student's *t*-test, d one-way ANOVA).

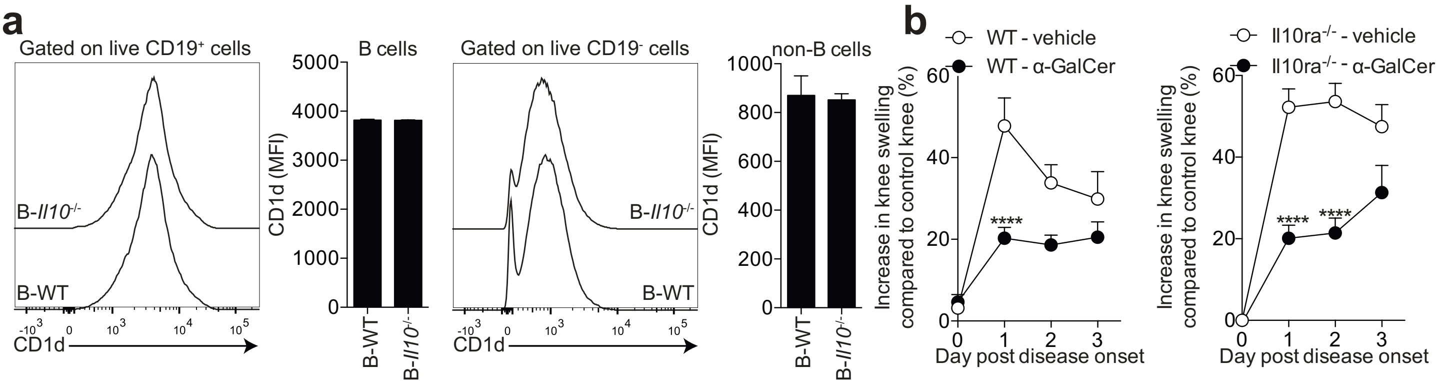

Supplementary Figure 5. CD1d expression in the absence of B cell IL-10. α-GalCer-mediated suppression of arthritis in IL-10Rα deficient mice. (a) Representative histograms and bar charts showing MFI of CD1d in splenic B cells (left) and non-B cells (right) from B-*Il10*$^{-/-}$ and B-WT mice (n=4 per group, one of two experiments is shown). (b) Mean clinical score of *Il10ra*$^{-/-}$ mice (right) and WT mice (left) that received α-GalCer or vehicle alone following induction of arthritis. Y axis shows percentage swelling in antigen-injected knee compared to control knee (*Il10ra*$^{-/-}$ n=6, WT n=8, one of two experiments is shown). a analyzed on day 3 post-disease onset. Data are mean±s.e.m. ****P<0.0001 (a Student's *t*-test, b two-way ANOVA).

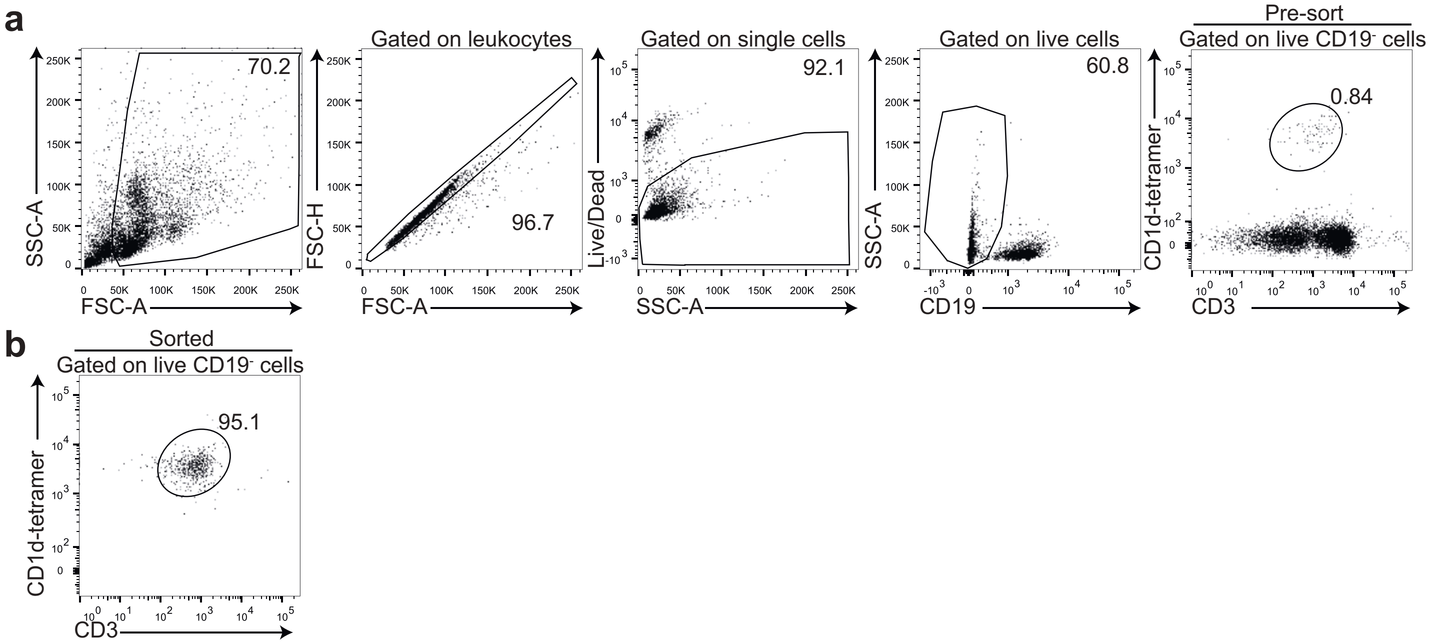

Supplementary Figure 6. Gating strategy and purity of iNKT cells. (a) Representative flow cytometry plots showing the sequential gating strategy for iNKT cells and iNKT cells prior to fluorescence activated cell sorting. (b) Representative flow cytometry plot showing iNKT cell purity following sorting. Used to gate and sort iNKT cells for RNA-seq in Figure 4.

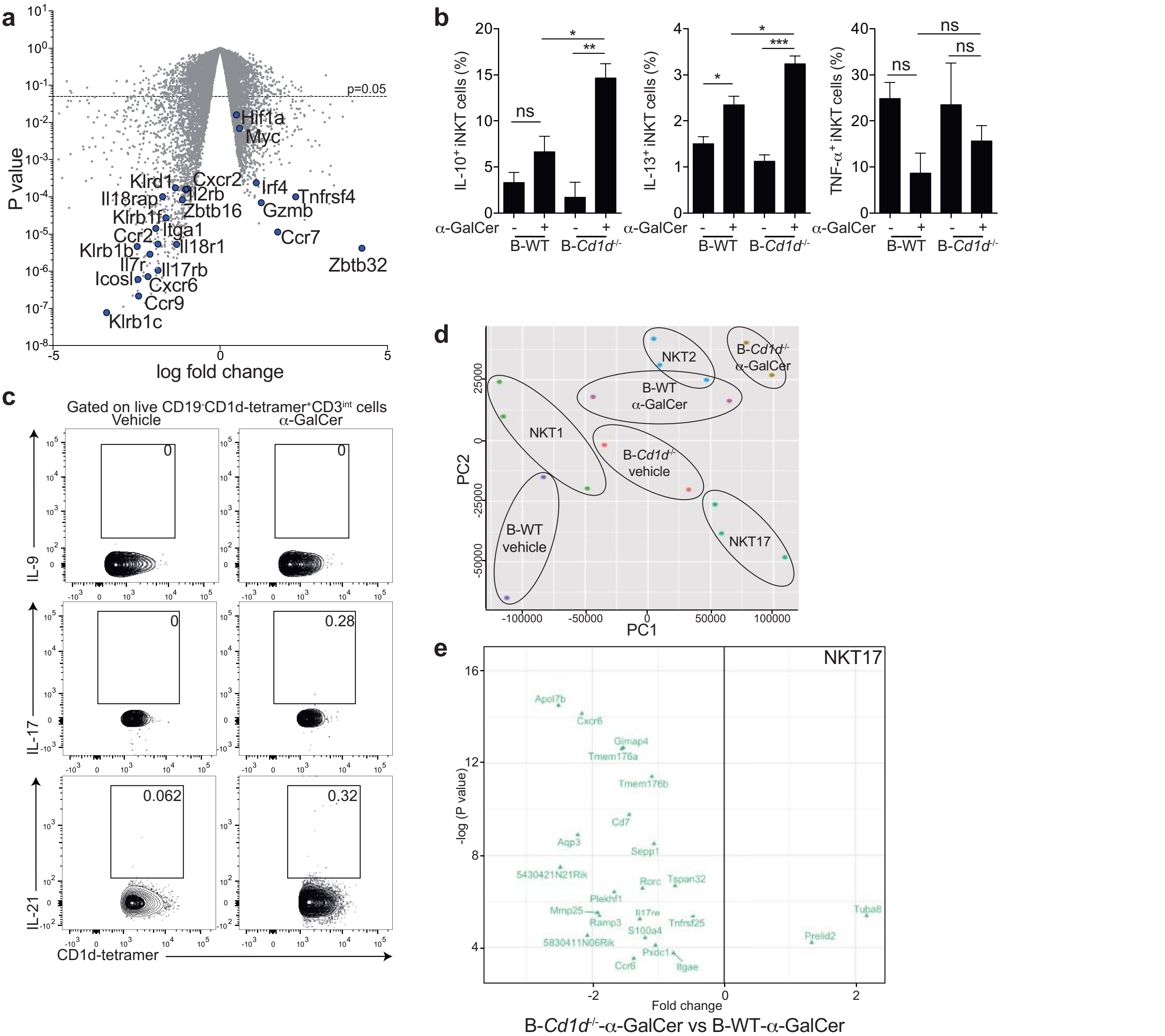

Supplementary Figure 7. B cell CD1d in iNKT cell gene expression. (a) Volcano plot showing fold changes of differentially expressed genes between iNKT cells from B-*Cd1d*$^{-/-}$ and B-WT chimeric mice following α-GalCer treatment, plotted against P values. (b) Bar charts showing the frequency of IL-10^{+}, IL-13^{+}, and TNF-α^{+} splenic iNKT cells from α-GalCer- or vehicle-treated B-*Cd1d*$^{-/-}$ and B-WT chimeric mice (n=3 per group, one of two experiments is shown). (c) Representative flow cytometry plots showing the frequency of IL-9^{+}, IL-17^{+}, IL-21^{+} splenic iNKT cells from α-GalCer- or vehicle-treated B-WT mice (one of three experiments is shown). (d) Principal component analysis of transcripts in iNKT cells from B-*Cd1d*$^{-/-}$ and B-WT chimeric mice that received α-GalCer or vehicle alone and published data sets for NKT1, NKT2 and NKT17 cells across experimental replicates. (e) Volcano plot showing fold changes of differentially expressed genes associated with NKT17 phenotype. a-e analyzed at 16h post-disease onset. Data are mean±s.e.m. ns not significant, **P*<0.05, ***P*<0.01, ****P*<0.001 (b one-way ANOVA).

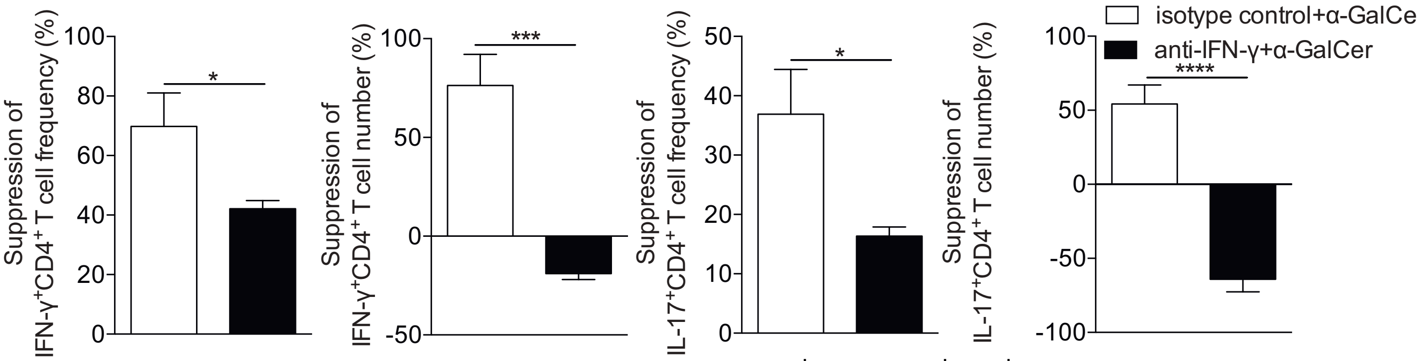

Supplementary Figure 8. Comparison of suppression of IFN-γ$^+$ and IL-17$^+$CD4$^+$ T cell responses by α-GalCer in anti-IFN-γ-treated and isotype-control-treated WT mice. Bar charts showing the suppression of IFN-γ$^+$CD4$^+$ (left) and IL-17$^+$CD4$^+$ (right) T cell frequency and number in anti-IFN-γ-treated and isotype-control-treated WT mice that received α-GalCer (n=3 per group, one of two experiments is shown). Analyzed on day 3 post-disease onset. Data are mean±s.e.m. *P<0.05, ***P<0.001, ****P<0.0001 (Student's t-test).

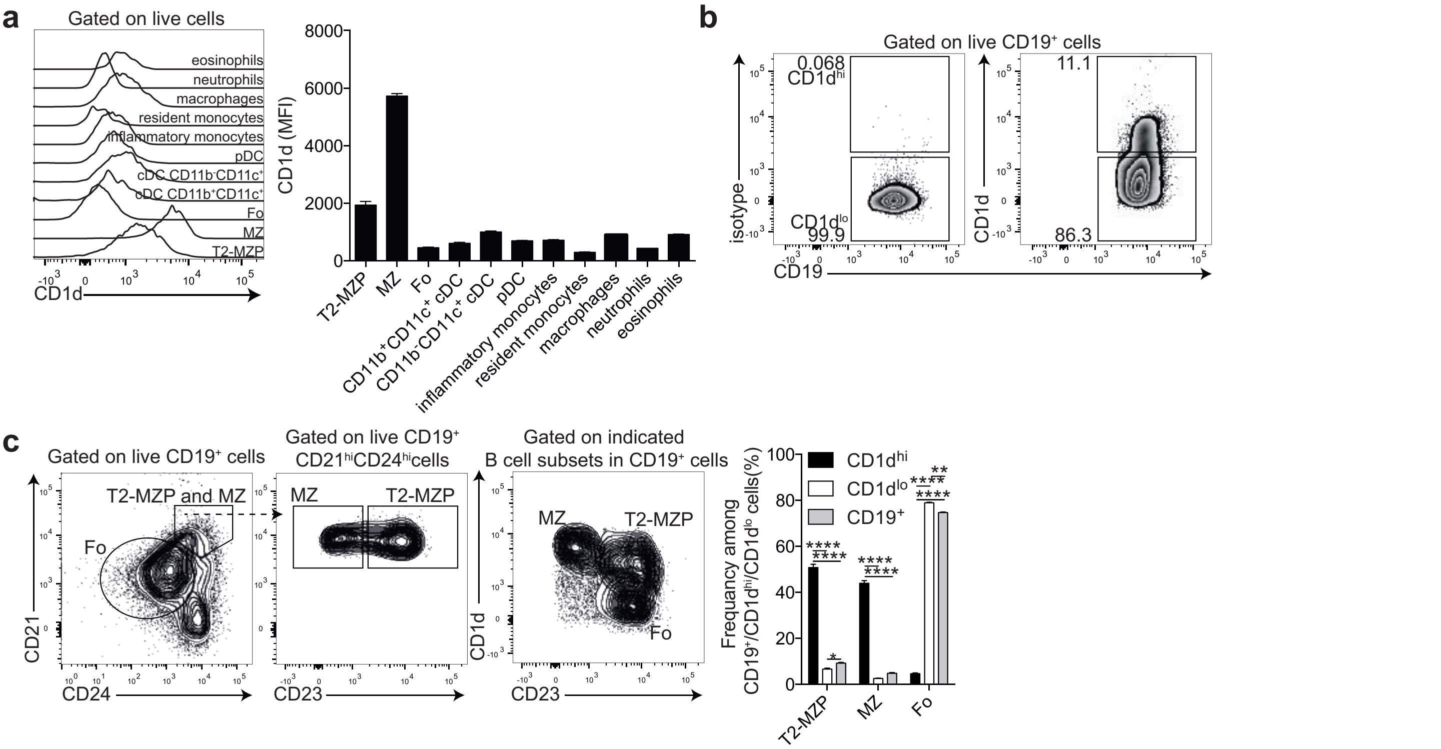

Supplementary Figure 9. Expression of CD1d by different antigen presenting cells. (a) Representative histograms and bar chart showing MFI of CD1d in antigen presenting cells in the spleens of WT mice (n=3 per group, one of four experiments is shown). (b) Representative flow cytometry plots showing the gating strategy to identify CD1d^{hi} and CD1d^{lo} B cells. (c) Representative flow cytometry plots showing the expression of CD1d in Fo, MZ and T2-MZP B cells in the spleens of WT mice, and bar chart showing the frequency of Fo, MZ and T2-MZP B cells in CD1d^{hi}CD19^{+}, CD1d^{lo}CD19^{+} or total CD19^{+} B cells in the spleens of WT mice (n=3, one of five experiments is shown). a-c analyzed on day 7 post-disease onset. Data are mean±s.e.m. ***P*<0.01, *****P*<0.0001 (c two-way ANOVA).

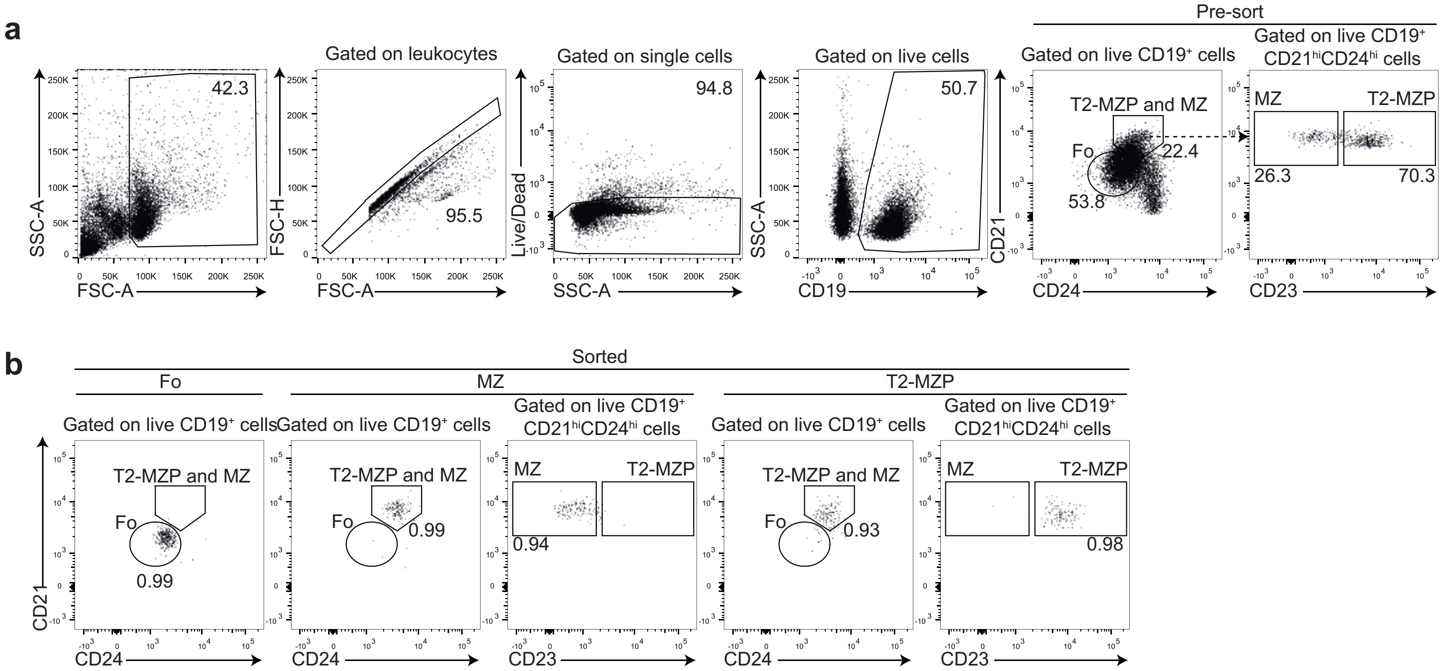

Supplementary Figure 10. Gating strategy and purity of B cell subsets. (a) Representative flow cytometry plots showing the sequential gating strategy for B cell subsets and B cell subsets prior to fluorescence activated cell sorting. (b) Representative flow cytometry plots showing B cell subset purity following sorting. Analyzed on day 7 post-disease onset. Used to gate and/or sort B cell subsets as in Figures 6, 7 and Supplementary Figures 9, 16 and 17.

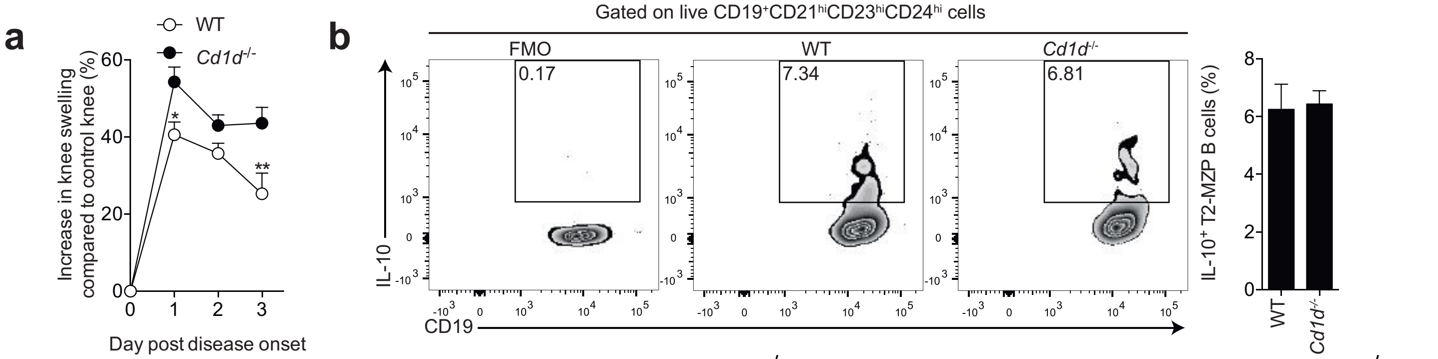

Supplementary Figure 11. AIA development in WT and *Cd1d*$^{-/-}$ mice. T2-MZP B cell IL-10 expression in *Cd1d*$^{-/-}$ and WT mice. (a) Mean clinical score of *Cd1d*$^{-/-}$ and WT mice following induction of arthritis. Y axis shows percentage swelling in antigen-injected knee compared to control knee (*Cd1d*$^{-/-}$ n=4, WT n=5, one of four experiments is shown). (b) Representative flow cytometry plots and bar chart showing the frequency of IL-10^{+} splenic T2-MZP B cells from *Cd1d*$^{-/-}$ and WT mice (n=5 per group, one of three experiments is shown). b analyzed on day 7 post-disease onset. Data are mean±s.e.m. **P*<0.05, ***P*<0.01 (a two-way ANOVA, b Student's *t*-test).

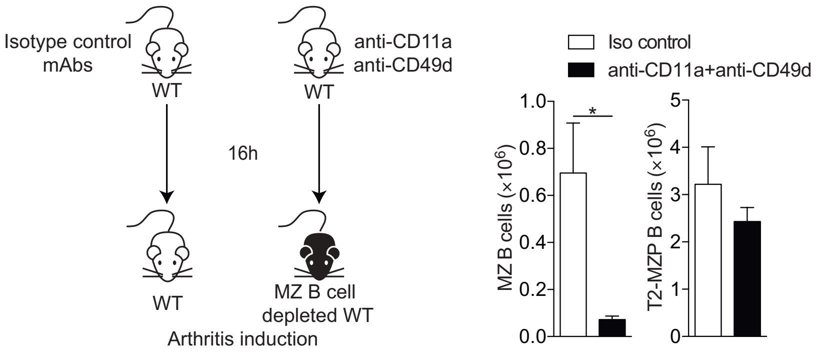

Supplementary Figure 12. Effect of anti-CD11a and anti-CD49d antibody treatment on MZ and T2-MZP B cells. Left, schematic showing experimental design. Right, bar charts showing the number of splenic MZ and T2-MZP B cells in anti-CD11a- and anti-CD49d-treated mice and isotype-control-treated mice prior to α-GalCer or vehicle administration (n=6 per group, one of two experiments is shown). Data are mean±s.e.m. *P<0.05 (Student's t-test).

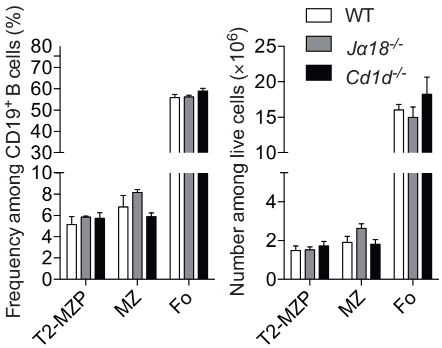

Supplementary Figure 13. Splenic B cell subsets in iNKT cell deficient mice. Bar charts showing the frequency and number of splenic T2-MZP, MZ and Fo B cells in *Jα18*$^{-/-}$, *Cd1d*$^{-/-}$ and WT mice (*Jα18*$^{-/-}$ n=6, *Cd1d*$^{-/-}$ n=7 and WT n=10, one of two experiments is shown). Data are mean±s.e.m. (two-way ANOVA).

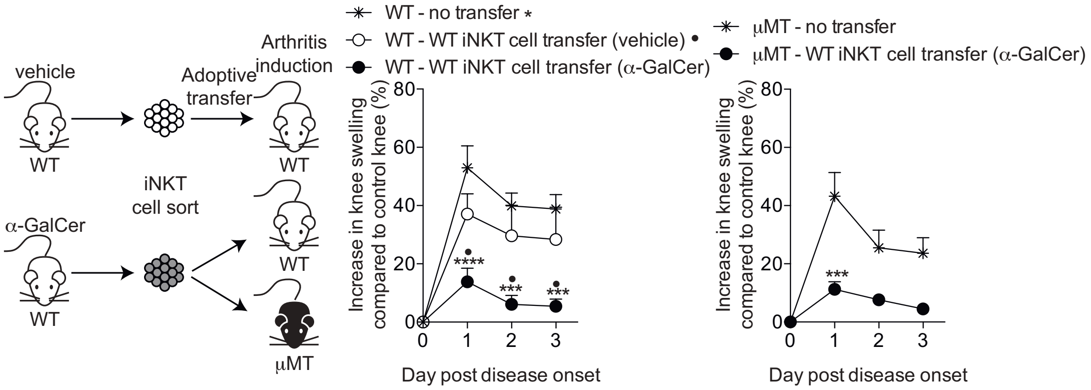

Supplementary Figure 14. iNKT cell suppressive capacity upon transfer to B cell deficient mice. Left, schematic showing experimental design. Middle, mean clinical score of WT mice that received iNKT cells isolated from the spleens of α-GalCer- or vehicle-treated WT mice on day 3 post-disease onset, or WT mice that received no transfer following induction of arthritis. Y axis shows percentage swelling in antigen-injected knee compared to control knee. Right, mean clinical score of µMT mice that received iNKT cells isolated from the spleens of α-GalCer-treated WT mice on day 3 post-disease onset, or µMT mice that received no transfer following induction of arthritis. Y axis shows percentage swelling in antigen-injected knee compared to control knee (n=4 per group, one of two experiments is shown). Data are mean±s.e.m. •*P*<0.05, ****P*<0.001, *****P*<0.0001 (two-way ANOVA).

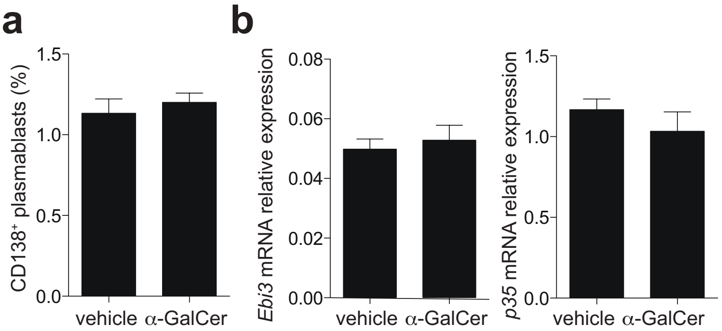

Supplementary Figure 15. CD138$^+$ plasmablasts and IL-35 following α-GalCer treatment. (a) Bar chart showing the frequency of splenic CD138$^+$ plasmablasts in α-GalCer- or vehicle-treated WT mice (n=3 per group, one of three experiments is shown). (b) Relative mRNA expression levels of *Ebi3* and *p35* in B cells isolated from α-GalCer- or vehicle-treated WT mice (n=3 per group, one of three experiments is shown). Data are mean±s.e.m. (Student's *t*-test).

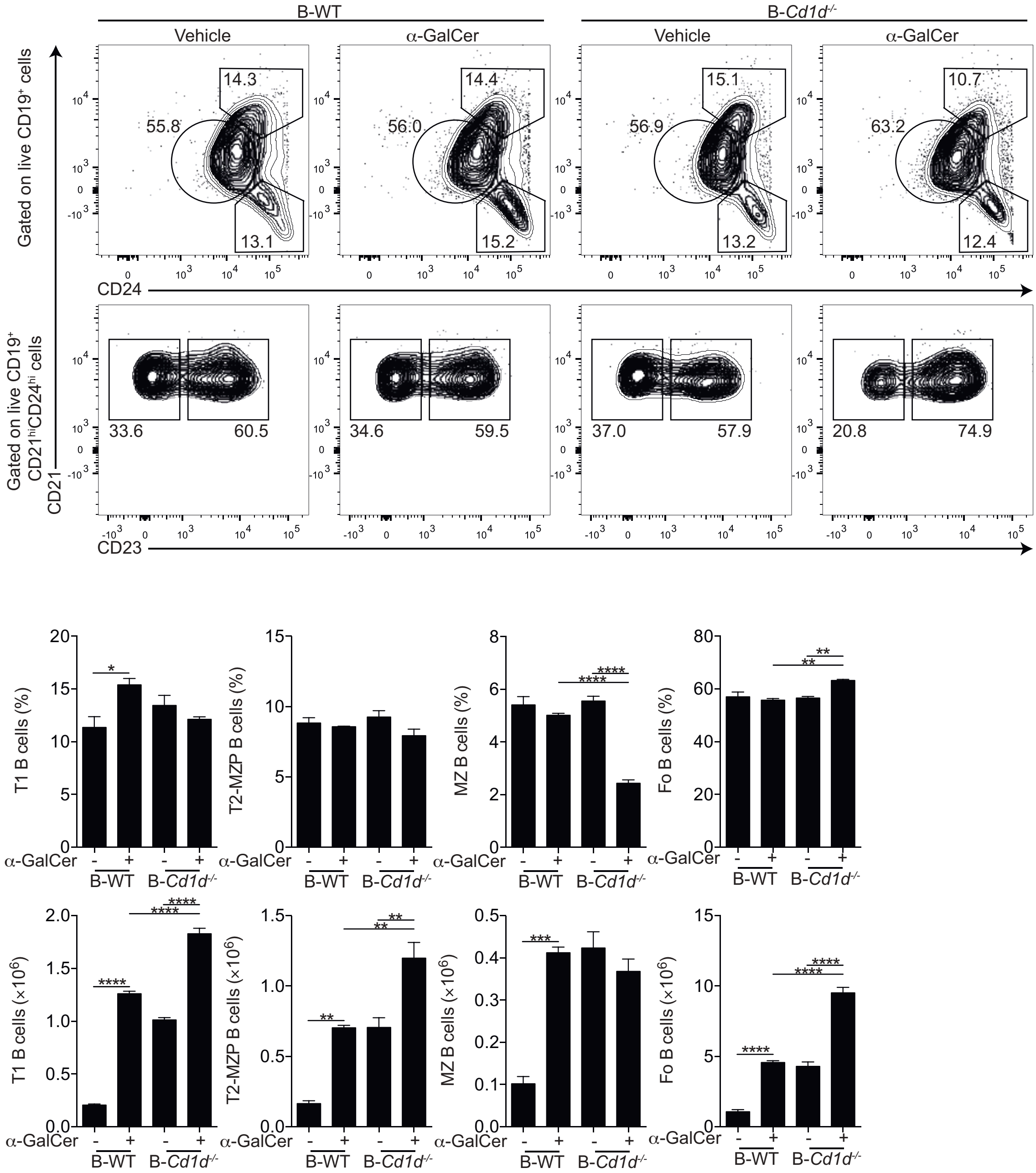

Supplementary Figure 16. Effect of α-GalCer treatment on splenic B cell populations. Representative flow cytometry plots (top) and bar charts (bottom) showing the frequency and number of splenic B cell subsets in B-*Cd1d*$^{-/-}$ and B-WT mice that received α-GalCer or vehicle alone (n=3 per group, one of two experiments is shown). Analyzed at 24h post-disease onset. Data are mean±s.e.m. **P*<0.05, ***P*<0.01, ****P*<0.001, *****P*<0.0001 (one-way ANOVA).

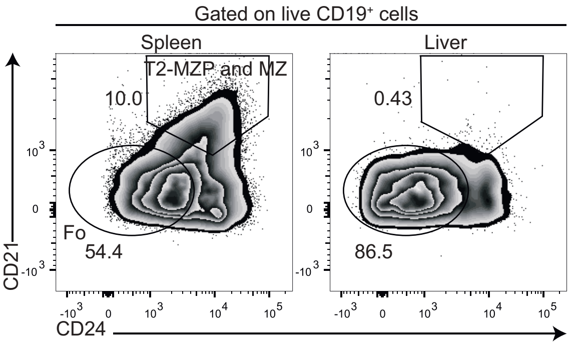

Supplementary Figure 17. Liver B cell populations. Representative flow cytometry plots showing the frequency of B cell subsets in the spleen (left) and liver (right) of WT mice (one of three experiments is shown).

Antibody	Clone	Dilution	Source
CD3-BUV395	145-2C11	1:200	BD Biosciences
CD3-PerCPCy5.5	17a2	1:200	BioLegend
CD4-BV711	RM4-5	1:200	BioLegend
CD8a-PECy7	53-6.7	1:200	BioLegend
NK1.1-BV421	PK136	1:200	BioLegend
NK1.1-PE	PK136	1:200	BD Biosciences
CD19-PECy7	1D3	1:200	BD Biosciences
CD19-PE	1D3	1:200	BD Biosciences
CD19-BV785	6D5	1:250	BioLegend
CD21-FITC	7G6	1:250	BD Biosciences
CD21-APC	7G6	1:250	BD Biosciences
CD21-PerCPCy5.5	7E9	1:250	BioLegend
CD23-PE	B3B4	1:250	BD Biosciences
CD23-BV605	B3B4	1:200	BD Biosciences
CD23-PECy7	B3B4	1:250	BioLegend
CD24-PerCPCy5.5	M1/69	1:250	BD Biosciences
CD24-APC	M1/69	1:250	BD Biosciences
CD24-BV421	M1/69	1:400	BioLegend
CD1d-PE	1B1	1:200	BD Biosciences
CD1d-PerCPCy5.5	1B1	1:200	BioLegend
CD1d-BV510	1B1	1:200	BD Biosciences
CD5-AF647	53-7.3	1:200	BioLegend
CD5-PE	53-7.3	1:200	BD Biosciences
IgD-PerCPCy5.5	11-26c.2a	1:200	BioLegend
IgD-BV510	11-26c.2a	1:200	BioLegend
IgD-BV711	11-26c.2a	1:200	BioLegend
IgM-APCCy7	RMM-1	1:200	BioLegend
IgM-FITC	RMM-1	1:200	BioLegend
B220-BUV395	RA3-6B2	1:200	BD Biosciences
CD11b-APC	M1/70	1:200	BioLegend
CD11b-APCCy7	M1/70	1:200	BioLegend
CD11b-BV711	M1/70	1:200	BioLegend

CD11c-PECy7	N418	1:200	BioLegend
CD11c-FITC	N418	1:200	BioLegend
I-A/I-E-PerCPCy5.5	M5/114.15.2	1:200	BioLegend
F4/80-BV605	BM8	1:200	BioLegend
F4/80-APC	BM8	1:200	eBioscience
Ly6C-BV785	HK1.4	1:200	BioLegend
Ly6G-BV421	1A8	1:200	BioLegend
Ly6G-BV605	1A8	1:200	BioLegend
Siglec F-PE	E50-2440	1:200	BD Biosciences
CD25-BV510	PC61	1:200	BioLegend
CD25-FITC	PC61	1:200	BioLegend
PLZF-PE	Mags.21F7	1:50	eBioscience
PLZF-PECy7	9E12	1:50	BioLegend
PLZF-PerCPCy5.5	9E12	1:50	BioLegend
Ki-67-PE	B56	1:50	BD Biosciences
Ki-67-PECy7	B56	1:50	BD Biosciences
IL-10-PE	JES5-16E3	1:50	BioLegend
IFN-γ-APC	XMG1.2	1:50	BD Biosciences
IFN-γ-FITC	XMG1.2	1:50	BD Biosciences
IL-17A-PE	TC11-18H10.1	1:50	BioLegend
IL-17-AF647	eBio17B7	1:50	eBioscience
TNF-α-APC	MP6-XT22	1:50	eBioscience
TNF-α-PE	MP6-XT22	1:50	BD Biosciences

Supplementary Table 1. Antibodies used for flow cytometry and cell sorting.
